# Food-Based Dietary Guidelines around the World: Eastern Mediterranean and Middle Eastern Countries

**DOI:** 10.3390/nu11061325

**Published:** 2019-06-13

**Authors:** Concetta Montagnese, Lidia Santarpia, Fabio Iavarone, Francesca Strangio, Brigida Sangiovanni, Margherita Buonifacio, Anna Rita Caldara, Eufemia Silvestri, Franco Contaldo, Fabrizio Pasanisi

**Affiliations:** 1 Epidemiology Unit, IRCCS Istituto Nazionale Tumori “Fondazione G. Pascale”, 80131 Napoli, Italy; 2 Internal Medicine and Clinical Nutrition, Department of Clinical Medicine and Surgery, Federico II University, 80131 Naples, Italy; lidia.santarpia@unina.it (L.S.); dr.fabioiavarone@gmail.com (F.I.); franstrangio@libero.it (F.S.); brigidasangiovanni@gmail.com (B.S.); margheritabuonifacio@libero.it (M.B.); arcaldara@libero.it (A.R.C.); miasilvestri@libero.it (E.S.); contaldo@unina.it (F.C.); pasanisi@unina.it (F.P.); 3 Interuniversity Center for Obesity and Eating Disorders, Department of Clinical Nutrition and Internal Medicine, Federico II University, 80131 Naples, Italy

**Keywords:** dietary guidelines, Eastern Mediterranean countries, healthy diet, non-communicable diseases, food safety, public health

## Abstract

In Eastern Mediterranean countries, undernutrition and micronutrient deficiencies coexist with overnutrition-related diseases, such as obesity, heart disease, diabetes and cancer. Many Mediterranean countries have produced Food-Based Dietary Guidelines (FBDGs) to provide the general population with indications for healthy nutrition and lifestyles. This narrative review analyses Eastern Mediterranean countries’ FBDGs and discusses their pictorial representations, food groupings and associated messages on healthy eating and behaviours. In 2012, both the WHO and the Arab Center for Nutrition developed specific dietary guidelines for Arab countries. In addition, seven countries, representing 29% of the Eastern Mediterranean Region population, designated their national FBDGs. At the moment several of these guidelines are available only in the English language. In summary, Eastern Mediterranean FBDGs mainly focus on food safety, not all are available in the local Arabic language, and they do not provide specific suggestions for the large number of foreign workers and migrants.

## 1. Introduction

Food and nutrition play a key role in the prevention and treatment of undernutrition and over-nutrition, diet-related non-communicable diseases (NCDs), such as obesity, cardiovascular diseases, diabetes, and some types of cancer [1,2]. In several Eastern Mediterranean countries, in the last few decades, several social health determinants such as political instability, low income, urbanization, demography, local conflicts, and migration have dramatically affected food availability and choices and consequently the nutritional status of certain populations [3,4]. In particular, inadequate intake of some nutrients is responsible for undernutrition and micronutrient deficiencies, whilst the increased consumption of processed (added sugars, saturated fat or trans fatty acids, NaCl- and calorie-rich) foods has played a key role in the increased incidence of NCDs [5,6,7,8,9,10,11,12,13,14]. Currently, two contrasting nutrition-related conditions co-exist: undernutrition and micronutrient deficiencies, especially among children, and overnutrition-related NCDs claiming over 2.2 million lives in 2012 and over 57% of all deaths in these countries [5,6,7,8]. In 2012, the Food and Agricultural Organization (FAO) and the World Health Organization (WHO), which pay particular attention to this issue worldwide, published Food-Based Dietary Guidelines (FBDGs) for the Eastern Mediterranean Region (EMRO) to provide the general population with indications for healthy nutrition and lifestyles. The aim of our study was to collect all the available national FBDGs of Eastern Mediterranean countries to identify differences and common points and to compare the suggested guidelines with European and American FBDGs.

## 2. Materials and Methods

All FBDGs from Eastern Mediterranean countries, as identified according to the WHO regional classification, were collected. The sources of information included the Internet, the FAO website, the Embassies cultural office in Italy and the National Ministries of Health of these countries. Countries with fewer than 100,000 inhabitants (according to the WHO website) were excluded. The data concerned the FBDG format, additional texts (such as leaflets, booklets providing further information and advice on the types and quantities to be consumed for each food group) and additional tips regarding fluids, alcohol, physical activity and body weight advice and individual healthy behaviours.

## 3. Results

### 3.1. Geographic Distribution of the Studied Countries

**Figure 1:**
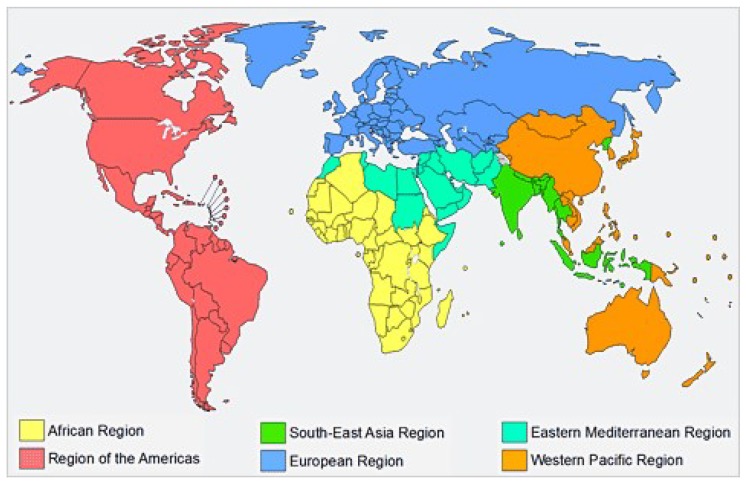
WHO Regions: WHO Member States are grouped into six regions. Each region has a regional office. The map shows the WHO regions and the location of the regional offices (https://www.who.int/about/regions/en/).

The WHO identifies 22 Eastern Mediterranean countries (Table 1, Figure 1), corresponding to a total of 684,561,000 inhabitants, about 9% of the world population [9].

Seven countries (Afghanistan, Iran, Yemen, Lebanon, Oman, Qatar and Saudi Arabia) have their own official FBDGs, reaching a total of 195,616,000 citizens (29% of the entire Eastern Mediterranean population) according to WHO documents [9]. Afghanistan published its national guidelines in 2015. Specifically, Afghanistan lacks a functioning healthcare system because it is one of the world’s most fragile and conflict-affected countries.

The WHO classification includes Iran, Afghanistan and Pakistan, which are not Arab countries, and does not include Algeria, which is an Arab country. Palestine, i.e., the territories of the Gaza Strip and West Bank, is included in the WHO EMR classification, whilst Turkey and Cyprus are considered to belong to the European Region, and have been discussed elsewhere [15]. All countries have more than 100,000 inhabitants. Twelve countries (Djibouti, Egypt, Jordan, Iraq, Libya, Morocco, Pakistan, Palestine, Somalia, Sudan, Syrian Arab Republic and Tunisia) had no specific official data, whilst Afghanistan, Iran, Lebanon, Oman, and Qatar (in the Near East region) have FBDG data available on the FAO website (Table S1). Food-based dietary guidelines were available for Saudi Arabia and Yemen on the National Ministries of Health website (Table S1). Our narrative analysis will start with a description of the general guidelines produced by the WHO in collaboration with the other UN agencies and experts from the region, aimed to guide the national guidelines: the “Promoting a healthy diet for the WHO Eastern Mediterranean Region (EMRO): user-friendly guide” [13]. Thereafter, the “Food Dome Dietary Guidelines for Arab Countries” [11], the “Food-Based Dietary Guidelines for Arab Gulf Countries” [12] and finally national guidelines, available for several countries, will be analysed to identify similarities and differences. Table 2 and Table 3 give a comparison of the dietary recommendations and non-dietary recommendations, respectively, for EMR countries’ FBDGs.

### 3.2. Promoting a Healthy Diet for the WHO EMRO: User-Friendly Guide

Based on the FAO/WHO Technical Consultation on National Food-Based Dietary Guidelines for countries in the Near East held in 2004, the WHO authored the “Promoting a healthy diet for the WHO EMRO: user-friendly guide” project to provide recommendations for an overall healthy pattern of eating to be adopted by the general population in EMRO countries to reduce the risk of major chronic diseases through diet and physical activity [13]. These recommendations are tailored to the dietary needs, food choices and preferences of the population of these regions and take into account the availability and cultural acceptance of foods in different countries. The guideline is available in English but not in the local language (Arabic), and, similarly to the USDA’s My Plate and Dietary Guidelines for Americans [16], a circle is used as a food guide pictorial representation. The plate is divided into five different sections, each representing a food group (bread, cereals, potatoes and rice; fruit and vegetables; meat, poultry, fish, dried beans and eggs; milk and dairy products; foods containing fat and foods and drinks containing sugar), with an area proportional to the recommended amounts to be consumed. A glass of water on the left of the plate encourages non-caloric fluid intake. A set of 14 recommendations (Table 4) suggests choosing a variety of healthy foods each day, describes the potential health benefits and negative disease outcomes related to each food category, and exhaustively discusses the recommendations for the respective food group.

A section on the glycaemic index and some tips to increase vegetable and fruit intake are included. It is suggested that people choose predominantly unsaturated vegetable oils (such as olive, sunflower, canola, corn and soy oils) rather than animal fats, lard, palm or coconut oil, hard margarine or clarified butter (ghee, samna). It is also recommended that people regularly consume legume-based dishes and at least two portions of fish per week to achieve adequate intake of omega-3 fatty acids, low-fat milk and dairy products, and to select lean meat cuts, with examples of local and traditional Arabic foods. The consumption of fresh or dried fruits (e.g., dates, apricots and raisins) as snacks instead of processed foods high in added sugars, of cereal-based snacks instead of cakes, biscuits, baklava, knafeh and confectionery and of fresh fruit juice instead of soft drinks and sweetened beverages (e.g., jellab, tamirhindi or sweetened lemonade) is suggested. Limits on added sugars, particularly in the form of sweetened beverages and sweets, salty snacks, and fatty foods are advised, while the importance of consuming dietary fat from unsaturated fat sources and omega-3 fatty acids from foods such as nuts, flaxseed and fish is emphasized. A list of foods and drinks containing added sugar is also included. Recommendations to consume less than 2.3 g of sodium per day and to use iodized salt for growth and brain development are included. Fresh foods are more frequently depicted than manufactured/packaged (processed) foods (such as yoghurt, butter, sweets, salty snacks, corn syrup and canned fish); oil is represented by a bottle. No recommendations on alcoholic beverage consumption are included. The WHO EMRO guidelines also provide advice to improve the nutritional status of specific population subgroups (hypertensive subjects, middle-aged and older adults, women of childbearing age, lactating women, strict vegans and vegetarians and lactose-intolerant individuals). These guidelines are intended for use not only by policy-makers, health care providers and nutritionists, but also by people involved in food distribution, food service and various nutrition programs. Unfortunately, they are not available in the Arabic language.

### 3.3. Food Dome Dietary Guidelines for Arab Countries

**Figure 2:**
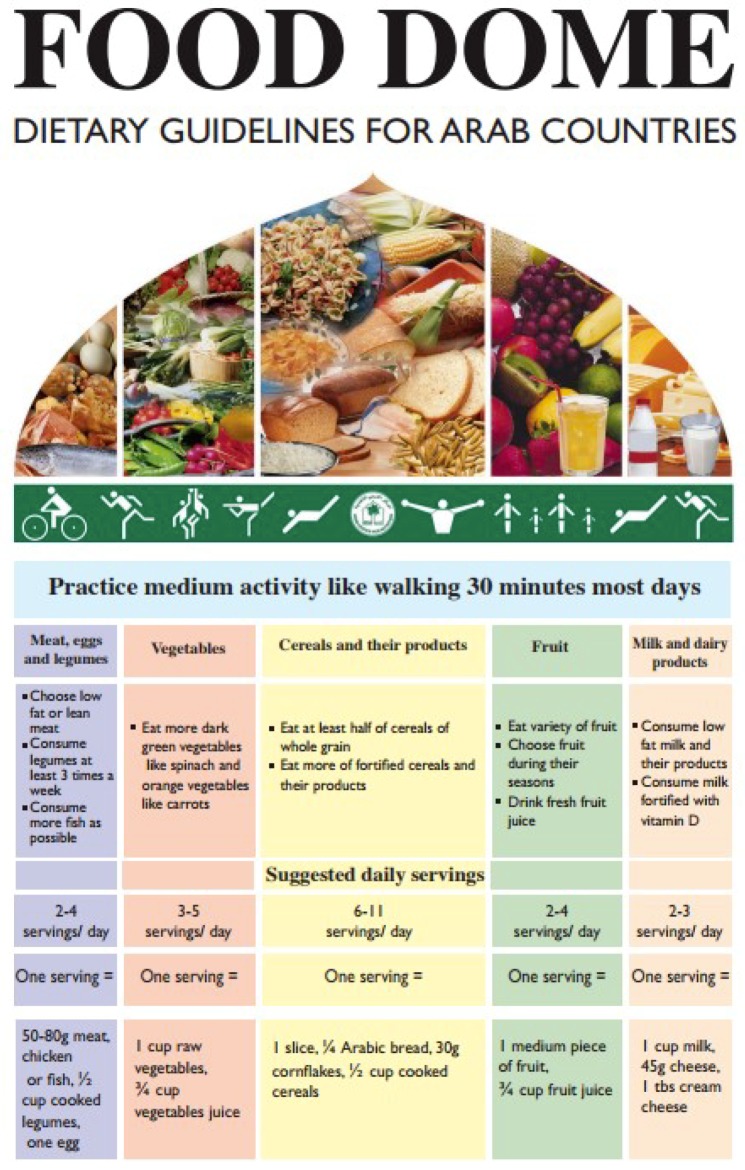
The Food Dome: dietary guidelines for Arab countries.

The Food Dome Dietary Guidelines proposed by the Arab Center for Nutrition [11] is focused on the prevention of the most prevalent diet-related diseases in Arabic countries and takes into account local habits and traditional food consumption patterns, lifestyle and health status. These guidelines are available both in the local language (Arabic) and in English. The Dome illustration used for the dietary guidelines reflects the culture and religious background of Arab people: the dome is part of most mosques and churches and is widely used in many buildings in the EMRO (Figure 2).

The Food Dome is divided into different sections, each representing a food group, proportional to the recommended amounts. A wide variety of foods commonly consumed by Arab people are represented, including traditional foods, such as Arabic flatbread and macaroni as cereals; cream cheese and laban in the milk and dairy food group; and seeds, nuts and peanut butter in the protein-rich food group. The graphical format is characterized only by fresh foods; milk is represented by a bottle. No recommendations on water and beverages, or on salt, sugar and fat intake, are reported. At the base of the Dome people are engaged in physical activity. In general, the Food Dome reflects the recommendations of promoting a healthy diet for the WHO EMRO: User-Friendly Guide [13] while adhering to regional and cultural food practices and access. Specific recommendations for vulnerable groups are also included: women of reproductive age, pregnant or lactating women, infants and preschool children (under six years of age), schoolchildren and adolescents, and people aged over 50 years. Advice is also included on alcohol consumption during pregnancy and its negative effects on children’s development and behaviour. Finally, recommendations for the prevention of cataracts and macular degeneration, and on adequate vitamin D intake and sunlight exposure to reduce risk factors for osteoporosis after menopause are included.

### 3.4. Food-Based Dietary Guidelines for Arab Gulf Countries

In 2012, the Arab Center for Nutrition developed Food-Based Dietary Guidelines for the Arab Gulf Countries (namely Bahrain, Kuwait, Oman, Qatar, Saudi Arabia, and United Arab Emirates, also part of the Gulf Cooperation Council (GCC) [12]. These countries are located in or connected to the Arabian Peninsula and have an overall population of nearly 50 million people, with the majority in Saudi Arabia (31 million) and the fewest in Bahrain (1.3 million).

The FBDGs for the Arab Gulf countries consist of 14 simple and practical recommendations, taking into account the socio-cultural status and nutritional problems shared by these countries. These guidelines lack a graphical representation and are available only in English [12]. It is suggested that people satisfy their energy requirements mainly from plant-related foods (grains, legumes, seeds, and nuts) and replace meat with fish wherever possible. It is also recommended that people eat grains fortified with iron, folic acid, calcium and vitamin D to compensate for deficiencies. The consumption of milk and low-fat dairy products is encouraged, particularly for their high calcium content and beneficial effects on bone density. Practical advice is also provided to reduce salt intake (no more than 5 g sodium chloride/day) and the consumption of salty food such as processed meat products (e.g., sausages and mortadella), salted fish and fish sauce, such as mihiyawa (mishawa) and tareeh, as well as giblets, due to their high cholesterol content. The use of spices, vinegar and herbs as salt substitutes, as well as of iodized salt, is recommended. Some practical advice to avoid contamination during food storage and/or preparation and alcohol abuse and smoking is also included.

### 3.5. Food Guide Illustration Shapes

**Figure 3:**
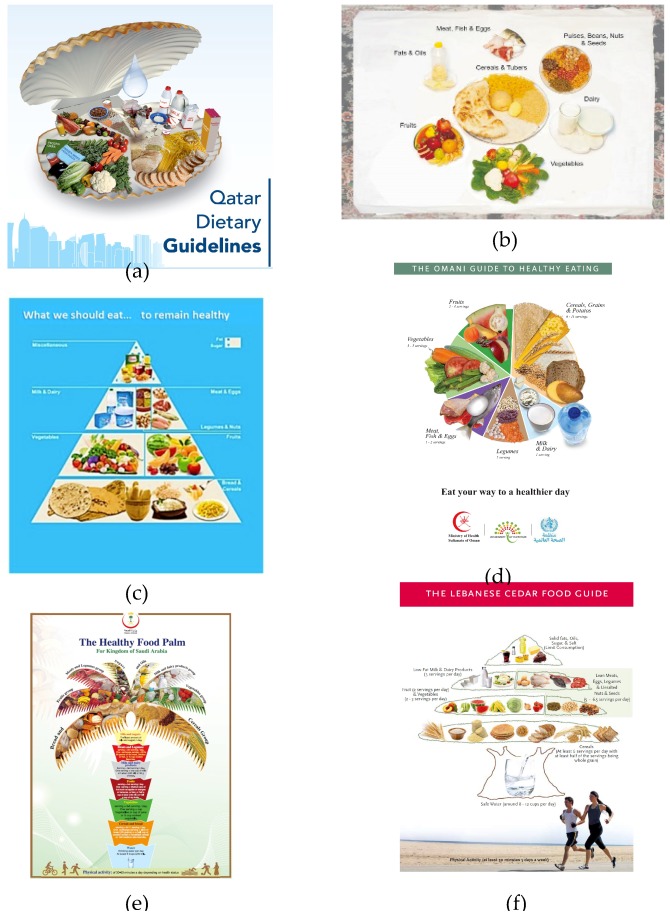
Food guide illustration shapes used for some Eastern Mediterranean FBDGs. (**a**) Qatar; (**b**) Afghanistan; (**c**) Iran; (**d**) Oman; (**e**) Kingdom of Saudi Arabia; (**f**) Lebanon.

In total, 6/7 (86%) EMR countries (Afghanistan, Iran, Lebanon, Oman, Qatar and Saudi Arabia) have visual aids for food guide illustrations (Figure 3).

Saudi Arabia, Iran and Lebanon use a pyramid as the food guide representation. The Iranian pyramid has four layers, accompanied by a list of 13 recommendations. The pyramid shape for Lebanon and Saudi Arabia adopts a graphical format that conveys aspects of local culture, like settings and crops. The Lebanese Cedar Food Guide has the crown/foliage shaped like a pyramid and is divided into six sections placed in four layers. Each section represents a food group, with the serving sizes according to the daily recommended amounts. A glass of water in the trunk of the cedar tree indicates the importance of consumption of safe water. A young man and a woman jogging on a beach suggest regular physical activity. The Lebanese manual “Fourteen Food-Based Dietary Guidelines for Lebanese Adults” deals with diet-related public diseases and the eating patterns of the Lebanese adult population. The Saudi Arabian “Healthy Food Palm” distributes food groups in the palm trunk (seven layers) and leaves according to the daily suggested amounts. At the bottom of the trunk a glass of water with recommendations on its consumption underscores the importance of water intake, mostly due to the very hot weather. Food groups on the leaves are symmetrically distributed according to the daily recommended amounts. At the base of the “Healthy Food Palm” people are shown playing football, swimming and cycling, recommending regular physical exercise. The palm tree symbolizes vitality, growth and prosperity and has a huge cultural influence in the Arab world; it is also part of the national flag of Saudi Arabia. Oman and Qatar use a circle as a food guide pictorial representation. The Omani Healthy Plate is the visual representation of the “Omani Guide to Healthy Eating.” The Omani FBDGs are set as nine Key Guidelines addressing people older than two years and focusing on adequate nutrition and NCD prevention. The plate is divided into six different coloured sections whose area is proportional to the recommended consumption. A bottle of water encourages non-caloric fluid intake. The Qatar food guide is a shell-shaped plate containing six food groups. The area of each food section is proportional to the recommended amount for a healthy diet; a drop of water symbolizes the importance of water consumption and hydration. Afghanistan uses a tablecloth with seven food plates. The largest plate at the centre represents the main food group, consisting of cereals and tubers.

### 3.6. Language Used

The Food Dome FBDGs for Gulf countries and the WHO EMRO guidelines were available only in English, whereas the Food Dome Dietary Guidelines for Arab countries were available both in the local language (Arabic) and in English. Five out of seven countries (Afghanistan, Iran, Lebanon, Oman and Saudi Arabia) have data and supportive information (web pages, leaflets and booklets) available in English, but not in the local languages. Qatari FBDGs are available both in the local language (Arabic) and in English.

### 3.7. Additional Information

Qatari FBDGs include ecological recommendations to protect the environment while eating a healthy diet; for example: reduce leftovers and waste; and choose fresh, home-made foods over highly processed foods and fast foods, preferably those produced locally and regionally. Due to possible microbial food contamination, all FBDGs include advice on food safety, proper cleaning practices and handling of food and contain additional text on personal hygiene measures. The Afghanistan, Lebanon and Oman FBDGs recommend using clean, safe water for hand washing, drinking and food preparation and suggest boiling water or using bottled water to avoid microbial and mineral contamination. In particular, the Omani FBDGs recommend not using untreated water from rivers and canals, as well as rainwater, which is prone to contamination. Recommendations on healthy cooking are present in the Iran, Lebanon, Oman, Qatar and Saudi Arabia FBDGs. Most FBDGs encourage consumers to check food labels to choose foods with fewer calories and low saturated fat (including trans fatty acids), sugar and sodium content. Five FBDGs (Afghanistan, Iran, Lebanon, Oman and Qatar) contain additional text on micronutrient intake for normal metabolic growth and physical well-being. Lebanon and Afghanistan suggest consuming fortified foods, such as vitamin D-fortified foods (e.g., milk and yoghurt), iron-fortified flour, vitamin A-fortified oil and iodine-fortified salt. Afghanistan FBDGs provide tables on the number of servings of each of the food groups needed for “three energy levels” to achieve and maintain a healthy body weight and overall health.

Five out of seven FBDGs recommend limiting the consumption of caloric beverages. Oman and Lebanon recommend limiting not only added-sugar soft drinks, to less than 10% of daily calories, but also sugar-free soft drinks. Some FBDGs recommend drinking natural fruit juice (e.g., orange, grapefruit, strawberry), a yoghurt drink or kefir instead of sweetened beverages and local syrup-based drinks (e.g., jellab, tamirhindi or sweetened lemonade).

### 3.8. Foods Pictured in the Graphics

Regarding the frequency of the food pictures represented in the FBDGs: cereals, fruit, vegetables, milk, dairy, fish, meat and legumes are reported in all countries (100%). In the analysed countries, fresh foods (85–98%) were depicted more frequently than manufactured/packaged foods (2–15%). Frozen okra and frozen mixed vegetables are also depicted in the Qatari shell food graphic because they are commonly consumed food products. Some countries (e.g., Lebanon, Oman, and Qatar) include local food preparations: Arabic bread and Arab flatbread, ghee (clarified butter), cheese, laban, Arab sweets and dates, among others. Water is part of the food graph for Lebanon, Oman, Qatar and Saudi Arabia, but absent in the Iran and Afghanistan FBDGs graphics. Salt consumption is graphically represented only in the Lebanese FBDGs, at the top of the cedar tree. Alcoholic beverages are absent in all graphic representations.

### 3.9. Food Grouping

The Lebanon, Saudi Arabia, Oman and Qatar FBDGs classify foods into six groups; water, generally is represented separately. “Milk and dairy products” are a food group in all FBDGs. Traditional dairy products, such as laban (a yoghurt-based drink), soy milk, kefir, Akkawi and Kashkaval (hard cheese), labneh and Kashta (a cooking cream cheese), are suggested. The Afghanistan and Lebanon FBDGs recommend the consumption of calcium- and vitamin D-enriched milk. The Qatari FBDGs include a “Milk, Dairy Products & Alternatives” food group that includes milk and dairy products and other calcium and vitamin D-rich foods (e.g., fortified soy drinks, almonds, chickpeas) as alternatives for people who do not drink milk or dairy products. Three countries (Afghanistan, Iran and Oman) classify animal (meat, fish and eggs) and plant-based protein-rich foods (legumes, seeds and nuts) as two different food groups. Some FBDGs include traditional meats, such as goat, sheep, rabbit, turkey, camel, lamb and liver as part of their food habits. The Omani FBDGs include animal protein-rich foods, both fresh and processed meat, and typical high-fat animal products (e.g., canned meats, sausages, shawarma—mixed meats placed on a vertical spit and grilled kebab, chicken nuggets and fingers). “Meats and Legumes” are reported as a unique protein-rich food group in the Saudi Arabian FBDGs. The Lebanese Food Guide lists fruit and vegetables by colour and nutrients: red for lycopene; orange and yellow for beta-carotene; green and purple for polyphenols; and white for allyl sulphides. The Omani FBDGs list fruit and vegetables by nutrients: vitamin C-, vitamin A-, iron- and folic acid-rich foods. In particular, they include the mulukhiya leaves of *Corchorus olitorius* in the iron/folic acid-rich vegetables group. The FBDGs of Afghanistan and Qatar recommend okra consumption (a local plant cultivated in tropical, subtropical and warm regions).

### 3.10. Salt Intake

Recommendations on salt intake are present in all FBDGs. The Afghanistan, Oman, Qatar and Lebanon FBDGs recommend no more than 5 g/day of salt, corresponding to 2.3 g/day sodium, whereas Saudi Arabia limits salt intake to less than 2.3 g/day. The Qatari FBDGs include additional information on how to check food labels for the words salt or sodium and distinguish “Foods high in salt” (more than 1.5 g of salt (0.6 g sodium)/100 g) and “Foods low in salt” (0.3 g of salt—or 0.1 g sodium—or less/100 g). The Saudi Arabia and Yemen FBDGs recommend using iodized salt, especially in cities that are not on the sea coast. The Lebanese FBDGs recommend limiting sodium intake to less than 2.3 g per day for healthy people and to less than 1.5 g for people with hypertension, type 2 diabetes, chronic kidney disease or over 50 years.

### 3.11. Lifestyle, Physical Activity and other Healthy Behaviours

Some FBDGs recommend maintaining a healthy body weight (6/7, 86%), eating a variety of foods, preferring vegetables to animal products (5/7, 71%), having a healthy breakfast (3/7, 43%), eating at regular times (2/7, 29%) and having some snacks based on fresh fruit and vegetables, unsalted nuts and seeds, whole cereal products or low-fat yoghurt (6/7, 86%). All FBDGs include physical activity as part of the format or as a key topic in the supporting information. Yemen FBDGs include the message: “Keep a better lifestyle: quit smoking and chewing qat.” Khat chewing is part of Yemeni culture, as well as in the Horn of Africa and the Arabian Peninsula, where the *Catha edulis* plant is widely cultivated. The chewing of khat leaves releases chemicals structurally related to amphetamines. Even if khat is not considered by the WHO a “seriously addictive drug,” its consumption can affect sleep, leading to rebound effects, such as late awakening, decreased productivity and daytime sleepiness, as well as increased heart rate and blood pressure. The Afghanistan, Lebanon, Oman and Qatar FBDGs include advice on sun exposure to maintain high vitamin D levels. Moreover, it is advised that people avoid excess sun exposure due to the risk of skin cancer.

### 3.12. Specific Population Subgroups

All EMR FBDGs include recommendations for the prevention of obesity, and some countries include recommendations for the prevention of diet-related diseases: CVD and hypertension (5 countries: Afghanistan, Iran, Lebanon, Oman, Qatar), diabetes (6 countries: Afghanistan, Iran, Lebanon, Qatar, Oman, Saudi Arabia), cancer (3 countries: Oman, Qatar, Iran) and dental caries (2 countries: Lebanon and Oman). The Qatar and Afghanistan FBDGs include advice on the prevention of obesity-related diseases, such as respiratory disease (BPCO), sleep apnoea, hernia, reproductive and mental health disorders. Moreover, the nutritional status of pregnant (Afghanistan, Lebanon, Oman, Saudi Arabia and Yemen) and breastfeeding women (Afghanistan, Lebanon, Oman, Qatar, Saudi Arabia, Yemen), as well as of children, adolescents (4/6) and the elderly (4/7), is considered. The Saudi Arabia and Afghanistan FBDGs include recommendations to prevent micronutrient deficiencies (such as iron, vitamins A and D, and iodine) in children. The Saudi Arabian FBDGs include recommendations to prevent osteoporosis and rickets. Specific dietary guidelines for vegetarians are also included in the FBDGs of Lebanon and Qatar; in addition, the Lebanese FBDGs include advice for strict vegans and for lactose-intolerant people.

## 4. Discussion

Eastern Mediterranean countries are experiencing a socioeconomic—either positive or negative—transition in health and nutritional status in the last decades [17,18]. In these regions, undernutrition and micronutrient deficiencies coexist with an alarming increase in obesity and NCDs associated with overnutrition [17,18,19,20]. Recently an International Commission has been instituted by *The Lancet* (The Lancet Commission) in order to regularly monitor and report on nutritional status around the world. The Commission’s last report [21] introduced the new concept of Global Syndemic to underscore the strict relationship between obesity, undernutrition and climate change. For example, in these countries, the epidemic of obesity is associated with iron deficiency anaemia and vitamin D deficiency, which, despite the sunny environment, remain two important nutritional issues and, in some countries, specific fortification policies are being considered. Many Arab governments have established a Nutrition Plan of Action for the prevention and control of nutrition-related diseases, as recommended by the WHO/FAO [19], but political instability, local persisting conflicts and migration make it difficult, or often impossible, to implement any nutritional or lifestyle advice.

According to the FAO and WHO recommendations, individual countries have developed simple dietary guidelines based on their specific public health concerns and relevant to people of different ages, lifestyles and cultures. Some countries (Oman, Qatar, Kingdom of Saudi Arabia) also detailed the process, involving many experts for development of FBDGs. Both Food-Based Dietary Guidelines for Arab Gulf Countries and Food Dome Dietary Guidelines for Arab Countries reported all steps for the Developing Food-Based Dietary Guidelines [11,12]. They described the review of their current nutrition problems and lifestyle patterns associated with diet-related diseases. Mainly, FBDGs focused on the remarkable economic and social transformations of the past few decades, which unavoidably influenced dietary habits.

Unfortunately, but understandably due to their instabilities, 12 out of 22 countries of this WHO region still do not have national FBDGs. Despite these limitations, Qatar is one of the few countries (along with Brazil, Germany and Sweden) to develop “environmentally sustainable and eating patterns that ensure food security, improve diet quality and respond to climate change challenges” [21,22].

### 4.1. Specific Nutritional Characteristics of EMRO FBDGs

The national food guidelines recorded in this review are comparable to the Mediterranean-style diet outlined by the WHO’s Regional Office for EMRO. These dietary recommendations focus on the predominant consumption of whole grains, fruits and vegetables and healthy plant-based oils, with a strong limitation on the consumption of red meats, animal-based proteins and fat, dairy products, added sugars and refined starchy foods. Indications on regular physical exercise, attention to food preparation, maternal health and diet during pregnancy and food hygiene support a healthy diet. No recommendations are given to limit the consumption of processed and ultra-processed (ready to heat, or ready to eat) foods, nor specific recommendations to avoid the excess consumption of added sugar drinks, in particular in younger groups. On the other hand, the suggestion to select vitamin- and mineral-rich or fortified foods is common. Another Arabic FBDG limitation that mainly regards the Gulf Cooperation Council is that, despite the prevalence of immigration, there is no specific advice targeted to the numerous foreigners living and working locally.

This may be an important limitation for multi-ethnic societies in this region of the globalized world. Migrants, in fact, represented approximately 51% of the region’s total population in 2016, ranging from 37% in Saudi Arabia to 89% in the United Arab Emirates [23].

EMRO FBDGs offer many practical pieces of advice that are appropriate for local customs, dietary patterns and daily lifestyles. Unfortunately, some of these FBDGs are only in English and not in the local language, Arabic. Several EMRO FBDGs are only available in English (Afghanistan, Iran, Lebanon, Oman, Saudi Arabia and Yemen), making their diffusion among middle- and lower-class populations quite difficult.

### 4.2. Special Recommendations Addressed to Local Environmental and Hygiene Peculiarities

The importance of drinking an adequate amount of safe water or fluids and their crucial role for proper hydration and thermoregulation are commonly reported in EMRO FBDGs because of the hot and dry weather typical of the region. Similarly, some FBDGs advise using clean and safe water for hand washing, drinking and food preparation, and caution against the use of untreated water from rivers, canals or rainwater collected in unprotected tanks. Microbial food poisoning (mainly caused by microbial agents, specifically salmonellosis, hepatitis A, shigellosis, and staphylococcus) is one of the most common food-borne diseases in these countries as a result of contaminated foods, a consequence of the diffuse practice of street food consumption. Nowadays about 20–25% people in developing countries (e.g., people with no access to cooking facilities because of rapid urbanization, single workers without families and people moving in and out of the city for work) depend on street food [4].

### 4.3. EMRO FBDGs Pictures and Food Grouping

The graphical representations used for FBDGs vary amongst EMR countries. The pyramid (Saudi Arabia, Iran and Lebanon) and the circle (Oman and Qatar) remain the most commonly used formats, while some other countries adopt icons inspired by national folklore and traditions. Moreover, by comparing these guidelines with the European and American ones, an inverse correlation seems to exist between industrialization, income and a simplified FBDG graphical representation. As a matter of fact, a number of countries are currently switching to the plate because it facilitates the interpretation of intake proportions of the food classes normally recommended. As far as food classes go, most FBDGs classify foods into six groups. The use of food groups ensures the inclusion, in separate “baskets,” of all basic foods and helps people make healthy food choices. In all countries, cereals, i.e., complex carbohydrates in different preparations, and vegetables occupy the largest proportion of the graphical representation. There is agreement regarding food grouping: minimal differences could be a result of the different emphasis given to the food nutritional properties or to local preferences and to the local food availability. “Fruits and Vegetables” are generally considered separate food groups in most countries (Afghanistan, Iran, Oman, Qatar and Saudi Arabia), but in the Lebanon FBDGs they are considered a single food group. In our opinion, fruit and vegetables should be represented separately, due to the different nutrient and caloric contents as well as the various distribution of vitamins, minerals and plant chemicals. Moreover, despite their indisputable protective roles against cancer, diabetes and cardiovascular diseases, the sugar content, in particular of fruit, must be carefully counted in the daily caloric intake.

### 4.4. Messages on Healthy Lifestyles

Associated with the FBDGs, messages on healthy lifestyles include, in the Yemeni FBDGs, the caution to not chew khat leaves (releasing chemicals structurally related to amphetamines) and in Gulf countries’ FBDGs the suggestion of “not smoking and reducing the exposure to smoking environments.” The smoking of Shisha (waterpipe tobacco smoking) is widely practiced by a considerable proportion of the population (also women and adolescents), and people incorrectly believe that this practice is not as harmful as smoking cigarettes. Alcoholic beverages are absent in all graphic representations, possibly for religious reasons, and no recommendation on alcohol consumption is included with the exception of the FBDGs for Arab Gulf countries, which include the rather specific recommendation “Avoid Drinking Alcoholic Beverages.” Nevertheless current economic open-market policies and globalization have contributed to a rise in local drinking of alcohol.

## 5. Conclusions

To our knowledge, this study is the only one to widely analyse several EMR countries’ FBDGs and compare them with European and American FBDGs. Specific features of Eastern Mediterranean and Middle Eastern countries’ FBDGs include separating vegetables from animal sources in designating protein-rich foods and preferring vegetable foods; attention to food safety and hygiene, particularly for street food and drinking water; a suggestion to consume iron-, calcium- and vitamin D-rich or fortified foods; and attention to tradition, inviting people to consume local foods and eventually reducing the caloric content of traditional recipes. More attention should be paid to ongoing ethnic, social and cultural evolution, giving adequate consideration to ethnic diversity.

## Figures and Tables

**Table 1:** EMR countries identified according to WHO classification and divided into six geographic sub-regions.

	North Africa (4)	Inhabitants (*n*)	Central East Africa (1)	Inhabitants (*n*)	Horn of Africa (2)	Inhabitants (*n*)	South Asia (3)	Inhabitants (*n*)	Western Middle East (5)	Inhabitants (*n*)	Arabian Peninsula (7)	Inhabitants (*n*)
												
	Egypt ^1^	92,115,000	Somalia ^1^	12,316,000	Sudan ^1^	40,783,000	Afghanistan	29,200,000	Syria ^1^	22,422,000	Saudi Arabia	32,552,000
	Libya ^1^	6,545,000			Djibouti ^1^	860,000	Pakistan ^1^	213,707,000	Lebanon	4,421,000	Yemen	27,426,000
	Tunisia ^1^	11,446,000					Iran	79,926,000	Jordan ^1^	10,053,000	Oman	4,560,000
	Morocco ^1^	34,852,000							Iraq ^1^	37,140,000	Un. Arab Emirates	9,121,000
									Palestine ^1^	4,706,000	Qatar	2,725,000
											Bahrain	1,501,000
											Kuwait	4,184,000
Total inhabitants per sub-region		144,958,000		12,316,000		41,643,000		322,833,000		78,742,000		82,069,000

^1^ No data available on FBDGs.

**Table 2:** EMR countries’ FBDGs dietary recommendations.

Food Groups	WHO‒EMR: User Friendly Guide	Food Dome Dietary Guidelines for Arab Countries	Kingdom of Saudi Arabia	Lebanese Dietary Guidelines	Qatar Dietary Guidelines	Omani Guide to Healthy Eating	Afghanistan	Islamic Republic of Iran
Cereals/Grain Products and Tubers	180 g/day: 90 g whole grain; 90 g “other” grains 30 g equivalent = 1 slice bread; ½ cup cooked pasta, rice, bulgar, or cereal; 1 cup dry cereal	6–11 servings/day: ≥5.5 servings whole grain 1 serving = 1 slice bread; ¼ Arabic bread; ½ cup cooked cereals; 30 g dry cereal	6–11 servings/day 1 serving = 1 slice bread (25 g), ½ cup of cereals, 1 slice of toast	6 servings/day (with at least ½ being whole grain) based on 2000 kcal diet 1 serving = ¼ big loaf of Arabic whole-wheat pita bread; 1 slice bread; ½ cup rice, pasta, or noodles; 1 cup dry cereal (unsweetened)	≥6 servings/day Substitute refined with whole and high-fibre grains. Choose grains prepared with little or no added fat, sugar or salt, read labels Avoid hydrogenated or trans-fat.	6–11 servings/day 1 slice bread; ½ cup cooked rice, pasta, or cereal; prefer whole wheat, brown rice	6 servings/day for a 2200 Kcal diet. 1 serving = ~140 kcal. ~ ¼ Naan (50 g piece); ~2/3 cup (125 g) cooked brown or white rice; 1 small potato boiled (160 g boiled weight), etc.	All types of bread (preferably whole), rice (brown, if available), macaroni, spaghetti, other pasta, barley
Fruits	4 servings/day or 2 cups/day 1 serving = 1 medium fruit; ½ cup fresh fruit; 1 cup fruit juice	3–5 servings/day 1 serving = 1 medium fruit; ¾ cup fruit juice	2–4 servings/day 1 serving = 1medium fruit; ½ cup juice; ½ cup dried fruit	2 servings/day 1 serving = 1 small fruit; 1 cup fruit juice; ½ cup dried fruit	2–4 servings per day 1 serving = 1 medium fruit; ½ cup cut fruit; ½ fruit juice; ¼ cup dried fruit. Favour whole fruit over juices, choose often as snacks	2–4 servings/day 1 serving = 1 cup raw or cooked; ½ cup fruit juice. Choose vitamin C-, vitamin A- and potassium-rich fruits.	3 servings/day for a 2200 kcal diet. 1 serving = ~80 kcal	Apples, pears, citrus fruit, peaches, grapes; dried fruits; fruit juices
Vegetables	5 servings/day or 2 ½ cups per day 1 serving = ½ cup raw or cooked; 1 cup leafy vegetable; ½ cup vegetable juice	3–5 servings/day 1 serving = 1 cup raw; ¾ cup vegetable juice	3–5 servings/day 1 serving = 1 cup raw or cooked; 1 cup juice	2–3 servings/day 1 serving = 1 cup raw or cooked; 2 cup leafy vegetables; 1cup vegetable juice	3–5 servings/day 1 serving = ½ cup cooked, fresh, raw, or canned; 1 cup green leafy vegetables	3–5 servings/day Chose vegetables prepared with little or no added fat and salt. Choose vitamin C-, vitamin A- and iron/folic acid-rich vegetables.	2.5 servings/day for a 2200 Kcal diet.1 serving = ~35 kcal	Green leafy and non-leafy vegetables
Milk & Dairy Products	3 cup equivalent/ day 1 cup equivalent = 1 cup low-fat milk or yoghurt; 45 g low-fat natural cheese; 60 g processed cheese; 8 tbsp labneh	2–3 servings/day 1 serving = 1 cup milk; 45 g cheese; 1 tbsp cream cheese	2–4 servings/day 1 serving = 1 cup milk or labneh; 30 g cheese	3 servings/day 1 serving = 1 cup low fat milk or dairy products to supply the daily recommended intake of calcium of 1000 mg/day based on 2000 kcal diet; 3 tbsp powdered milk; 45 g cheese; 8 tbsp labneh	2 cup equivalents/day 1 cup eq. = 1 cup milk or yoghurt; 50 g cheese; 14 tbsp labneh Daily consumption of r low fat milk and dairy products. Choose vitamin D fortified milk	1 serving/day 1 serving = 1 cup long-life, fresh, pasteurized, powdered milk or yoghurt; 45 g natural cheese; 60 g oz processed cheese, laban and kushk	3.5 servings/day for a 2200 Kcal diet. 1 serving = ~70 kcal	Milk, cheese, yoghurt, yoghurt drink (doogh), kashk (a traditional dry milk product), ice cream
Meat & Vegetal Proteins	160 g per day 1 serving = 30 g lean meat, poultry, or fish; 1 egg; ¼ cup cooked dry beans; 15 g nuts or seeds	2–4 servings per day 1 serving = 50–80 g meat, chicken, or fish; 1 egg; ½ cup legumes and nuts	2–3 servings per day 1 serving = 60–90 g red meat, chicken, or fish; ½ cup cooked legumes	5–6.5 servings per day 1 serving = 30 g meat, poultry, or fish; 1 egg; 1 cup legumes; 15 g nuts or seeds	Eat a variety of fish at least 2 times a week. Chose skinless poultry and lean cuts of meat. Avoid processed meats. Chose legumes, nuts and seeds as alternative protein sources. Eat legumes daily. Choose legumes prepared with little or no added fat or salt	Meat: 1–2 servings/day 1 serving = 30 g red lean beef, lamb and camel, poultry, chicken. All fishes; 1 egg; 15 g oz nuts or seeds Legumes: 1 serving per day; ½ cup cooked lentils, beans or peas; ¼ cup cooked dry beans or tofu	Meat: 2 servings/day for a 2200 Kcal diet. 1 serving = ~70 kcal. Legumes: 1.5 servings/day for a 2200 Kcal diet. 1 serving = ~140 kcal.	Beef, veal, lamb, chicken, fish, canned tuna, shrimp, eggs Legumes, nuts including walnuts, almonds, pistachios, peanuts, hazelnuts
Oils	6 tsp per day	None provided	“least amount per day”	Limited consumption	Limited Consumption	None provided	None provided	None provided
Salt, Fats & Sugars	Salt: no more 2,3 g/day. Use iodized salt for growth and brain development Fat: 18 g/day Sugar: 8 tsp/day	None provided	Use iodized salt, especially in cities that are not on the sea coast “Least amount per day”	Salt: no more 2.3 g/day for healthy people and to less than 1.5 g for people with hypertension, type 2 diabetes, chronic kidney disease, or over 50 years. Fat: 56–78 g per day Sugar: <10 tsp per day	Salt: <5 g/day Additional information on how to check food labels for the words salt or sodium. Distinguish “Foods high in salt” and “Foods low in salt” Fat: <3 g per 100 g Sugar: <5 g per 100 g	Salt: <5 g/day; Fat: 59 g per day Sugar: <10% total calories sugar	Salt: <5 g/day; Reduce simple sugars and substitute sweets with fruits. Remove visible fat from meat. Reduce processed meat consumption.	None provided
Water & Fluids	Men: 3.7 L/day Women: 2.7 L/day	“Sufficient quantity”	1.5 L per day	2–3 L per day	2–3 L per day	Daily	Daily	Daily

Tbsp: tablespoon; tsp: teaspoon.

**Table 3:** EMR countries’ FBDGs non-dietary recommendations.

Non Dietary Recommendations	WHO EMRO: User Friendly Guide	Food Dome Dietary Guidelines for Arab Countries	Kingdom of Saudi Arabia	Lebanese Dietary Guidelines	Qatar Dietary Guidelines	Omani Guide to Healthy Eating	Afghanistan	Islamic Republic of Iran
Physical Activity	30 min/day of moderate PA	30 min/day of moderate PA	30–60 min/day	30 min, 5 days a week	30 min moderate PA, 5 days a week	Moderate PA: 30 min 5 days/week, Vigorous PA:20 min 3 days/week	20–30 min PA/day	30–40 min PA/day
Language	English	English, Arabic	English	English	English, Arabic	English	English	English
Food guide illustration shape	Plate (circle)	Food Dome	Healthy Food Palm	Lebanese Cedar (pyramid)	Tablecloth	Healthy Plate (circle)	Tablecloth	Pyramid
Food safety/Hygiene	Five keys for safer foods. Eat clean and safe food.		Ensure Safety of Food Eaten	Proper cleaning practices and food handling. Microbiological aspects of food safety, and practical matters related to safety precautions	Mothers and family members should practice hand washing-with soap and water at critical times	Wash your hands before handling food and often during food preparation, after going to the toilet. Wash and sanitize all surfaces and equipment used for food preparation. Protect kitchen areas and food from insects, pests and other animals	Mothers and family members should practice hand washing with soap and water at critical times	Washing hands and keeping chopping boards, plates, knives, etc. clean
Safe water	Drink lots of clean water			Get your home tap water checked for microbial and mineral contamination. If it is not safe for drinking, drink safe bottled-water	Use clean and safe water for hand washing, drinking and food preparation	Untreated water from rivers and canals is not safe! Rainwater collected in clean tanks is safe as long as the tanks are protected from contamination from birds or other animals	Use clean and safe water for hand washing, drinking and food preparation	
Healthy body weight	Maintain a healthy body weight	Maintain proper weight for height	Maintain an appropriate weight for your height	Enjoy and maintain a healthy body weight		Exercising regularly can help maintain a healthy body weight and high quality of life		Maintain a normal weight and stay healthy; you should eat adequately and have sufficient physical activity
Recommendations for specific population subgroups	Women of childbearing age, lactating women, strict vegetarians, lactose intolerance, elderly	Pregnant and lactating women, infants and preschool children, school children and adolescents, people aged 50 years		Pregnant, breastfeeding women, menopause, elderly, lactose intolerant, Vegetarians and strict vegans. Population groups most susceptible to food-borne illnesses (individuals with weakened immune systems, e.g., HIV-infected)	Pregnant, breastfeeding women, children, adolescents, vegetarians	Pregnant, breastfeeding women, children, adolescence, elderly	Pregnant, breastfeeding women, children, adolescence	
Recommendations for specific diseases	Coronary heart disease, stroke, cancers, type 2 diabetes mellitus, cataract and macular degeneration, hypertension; dental caries	Diet-related diseases (heart disease, type 2 diabetes, hypertension, osteoporosis, obesity and cancer), undernutrition and micronutrient deficiencies	Obesity; CVD; hypertension, diabetes, dental caries, osteoporosis, rickets, micronutrient deficiencies	Obesity; CVD; diabetes, hypertension, obesity, cancer, dental caries, osteoporosis, nutrient deficiencies	Obesity; CVD; diabetes, hypertension, cancer, nutrient deficiencies, COPD	Obesity; CVD; hypertension, diabetes, obesity, cancer, dental caries, osteoporosis, nutrient deficiencies	Obesity; CVD; hypertension, BPCO, micronutrient deficiencies	Obesity; CVD; hypertension, diabetes, cancer, nutrient deficiencies

PA: Physical Activity; CVD: Cardiovascular Diseases; COPD: Chronic obstructive pulmonary disease.

**Table 4:** Promoting a healthy diet for the WHO Eastern Mediterranean Region: user-friendly guide—key recommendations.

1.	Maintain a healthy body weight
2.	Be active
3.	Limit intake of fats and oils
4.	Limit intake of sugars, especially sweetened foods and beverages
5.	Limit salt intake
6.	Eat a variety of foods every day
7.	Eat cereals, preferably whole grains, as the basis of most meals
8.	Eat more vegetables and fruit every day
9.	Eat legume-based dishes regularly and choose unsalted nuts and seeds
10.	Eat fish at least twice a week
11.	Consume milk/dairy products daily (preferably low-fat)
12.	Choose poultry and lean meat
13.	Drink lots of clean water
14.	Eat clean and safe food

## Supplementary Materials

The following are available online at https://www.mdpi.com/2072-6643/11/6/1325/s1, Table S1: Food-Based Dietary Guidelines sources for EMR countries.
