# Supplementary material for: Food-Based Dietary Guidelines around the World: Eastern Mediterranean and Middle Eastern Countries
Source: Nutrients. 2019 Jun 13;11(6):1325. doi: 10.3390/nu11061325 (PMC6627223; doi:10.3390/nu11061325)
Supplement: Supplementary file 1 [file nutrients-11-01325-s001.pdf]

Supplementary Table 1. Food-Based Dietary Guidelines sources for EMR countries.

Countries	Sources
Afghanistan	http://nutritionmoph.gov.af/attachments/article/92/SOP%20for%20Public%20Nutrition%20English.pdf; http://www.fao.org/nutrition/education/food-dietary-guidelines/regions/countries/afghanistan/en/
Iran	http://www.fao.org/nutrition/education/food-dietary-guidelines/regions/countries/iran/en/
Lebanon	http://www.fao.org/nutrition/education/food-dietary-guidelines/regions/countries/lebanon/en/
Oman	http://www.fao.org/nutrition/education/food-dietary-guidelines/regions/countries/oman/en/
Qatar	http://www.fao.org/nutrition/education/food-dietary-guidelines/regions/countries/qatar/en/
Saudi Arabia	http://www.moh.gov.sa/en/HEALTHAWARENESS/Pages/SaudihealthFoodGuide.aspx; http://www.moh.gov.sa/endepts/Nutrition/Pages/Palmfood.aspx
Yemen	http://www.cmamforum.org/Pool/Resources/YEMEN-National-Nutrition-Strategy-2009.pdf
